# Establishment of a Genetic Transformation and Gene Editing Method by Floral Dipping in *Descurainia sophia*

**DOI:** 10.3390/plants13202833

**Published:** 2024-10-10

**Authors:** Tianjiao Jia, Hua Yang, Dingding Zhou, Sanzeng Zhao, Jianyong Wang, Tao Zhang, Mingkun Huang, Danyu Kong, Yi Liu

**Affiliations:** Lushan Botanical Garden, Jiangxi Province and Chinese Academy of Sciences, NO.9 Zhiqing Road, Jiujiang 332900, China; jiatj@lsbg.cn (T.J.); yangh@lsbg.cn (H.Y.); zhoudingdiing@163.com (D.Z.); zhaosanzeng@163.com (S.Z.); wangjianyong90@163.com (J.W.); zhangt@lsbg.cn (T.Z.); huangmk@lsbg.cn (M.H.); kongdy@lsbg.cn (D.K.)

**Keywords:** *Descurainia sophia*, floral dip method, genetic transformation system, CRISPR/Cas9

## Abstract

*Descurainia sophia* L. Webb ex Prantl is used in traditional medicine globally. However, the lack of an efficient and reliable genetic transformation system has seriously limited the investigation of gene function and further utilization of *D. sophia*. In this study, a highly efficient, time-saving, and cost-effective *Agrobacterium tumefaciens*-mediated genetic transformation system has been developed in *D. sophia*. In this method, the transformation was accomplished by simply dipping developing *D. sophia* inflorescences for 45 s into an *Agrobacterium* suspension (OD_600_ = 0.6) containing 5% sucrose and 0.03% (*v*/*v*) Silwet L-77. Treated plants were allowed to set seeds which were then plated on a selective medium with hygromycin B (HygB) to screen transformants. Additionally, the CRISPR/Cas9 genomic editing system was validated by targeting phytoene desaturase (*PDS*) gene using this floral dip method, and mutant plants with the expected albino phenotype could be obtained in 2.5 months. This genetic transformation and targeted editing system will be a valuable tool for routine investigation of gene function and further exploitation in *D. sophia*.

## 1. Introduction

*Descurainia sophia* L. Webb ex Prantl, an annual dicot weed belonging to Brassicaceae family, has been used in traditional medicine globally because of its important pharmaceutical values [1,2]. *D. sophia* is a diploid, self-pollinating plant with a height ranging from 60 to 80 cm. The flowering process of *D. sophia* starts approximately 1.5 months after germination, and the period of seed to seed takes around 2 months in our laboratory conditions. It exhibits remarkable reproductive and growth potential in diverse environments, yielding about 4000–10,000 seeds per plant. To date, the genome sequence of *D. sophia* remains unreported. Various compounds have been isolated from this plant, such as phenolic compounds (flavonoids and coumarins), fatty acids, lipids, and steroids, with diverse biological effects, which include anti-inflammatory, anti-oxidative, antipyretic, anthelmintic activities, etc., [3,4,5,6,7]. Additionally, many studies have proven its effects on cancer, respiratory, gastrointestinal, and cardiac systems, without any significant adverse effects [2,3,8,9]. Moreover, *D. sophia* also has potential use as an oil crop because of its high productivity, high seed oil content up to 44.1%, and unique fatty acid composition, which mainly consists of linolenic acid and palmitic acid having biological effects within the organism [8,10]. *D. sophia* has successfully attracted the attention of researchers from different countries, as a valuable medicinal plant and oil crop. In the past decades, substantial progress of *D. sophia* has been experienced on chemical composition analysis and pharmacological mechanism [3,4,5,6,7,11,12,13]. However, the lack of an efficient transformation protocol for *D. sophia* severely hinders the fundamental research and further utilization.

Plant genetic transformation provides a range of opportunities for basic research and genetic modification of valuable traits in plants of economic importance. *Agrobacterium*-mediated transformation remains the most frequently used approach for the introduction of transgenes into Brassicaceae [14]. As a classic *Agrobacterium*-mediated transformation method, the floral dip method is simple, convenient, stable, and inexpensive. This method was first established in *Arabidopsis thaliana* and has been successfully utilized in other Brassicaceae plants, such as *Brassica rapa*, *B. napus*, and *B. carinata* [15,16,17]. Possibly, it could be utilized in *D. sophia*, which belongs to the Brassicaceae family. CRISPR/Cas9 is a powerful tool for precise genome editing and broadly used to modify metabolic pathways and develop plants with optimized secondary metabolite profiles in many medicine plants, such as *Dioscorea zingiberensis*, *Salvia miltiorrhiza*, and *Symphytum officinale* [18,19,20,21,22].

In this study, we established an *Agrobacterium*-mediated genetic transformation system for *D. sophia* via the floral dip method. Additionally, the targeted genome editing was also successfully accomplished in *D. sophia*, using the transformation method developed in this study. The genetic transformation and targeted editing system presented here will significantly advance basic research and utilization of *D. sophia* in the near future.

## 2. Results and Discussion

**Figure 1:**
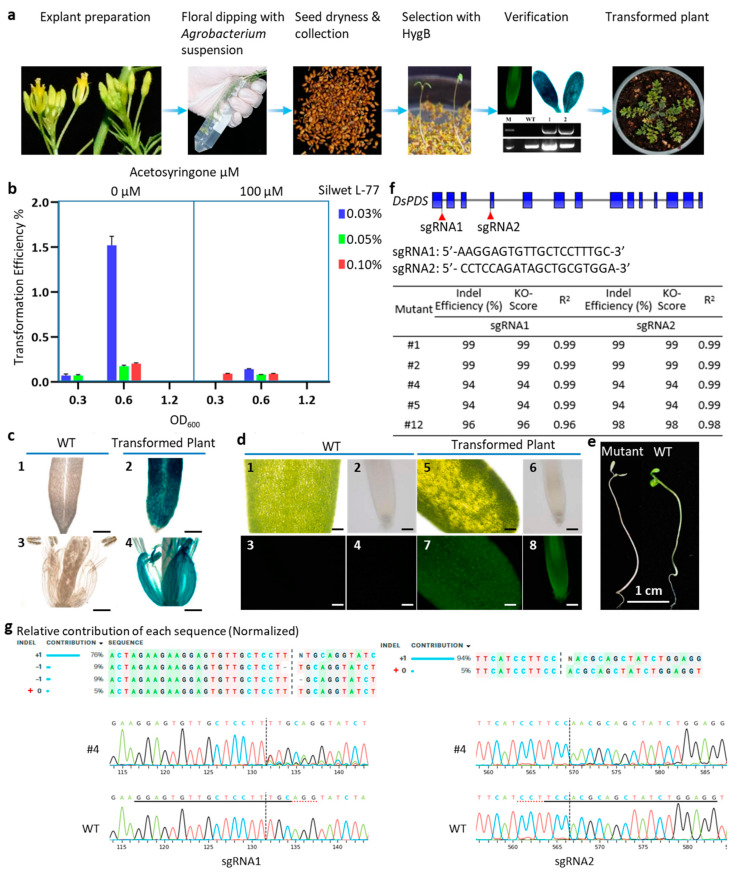
***A. tumefaciens*-mediated transformation and gene editing of** *D. sophia* **with the floral dip method.** (**a**) Schematic of *Agrobacterium*-mediated transformation of *D. sophia* by the floral dip method. (**b**) Transformation efficiencies for *D. sophia* under different OD_600_ values of *Agrobacterium*, AS concentrations, and Silwet L-77 concentrations. All seeds yielded by each plant have been sown on the selection plates in this assay. Data are mean ± SD. (**c**) GUS staining analysis of leaf and flower from WT *D. sophia* plants (1, 3) and T_1_ transgenic plants (2, 4). Bar: 400 μm. (**d**) GFP analysis of WT and transgenic *D. sophia* plants. The photos of leaf and root from WT *D. sophia* plant (1, 2) and T_1_ transgenic seedling (5, 6) were taken under white light, respectively. The photos of leaf and root from WT *D. sophia* (3, 4) and T_1_ transgenic seedling (7, 8) were taken in fluorescence, respectively. Bar: 100 μm (1, 3, 5, and 7), 50 μm (2, 4, 6, and 8). (**e**) Representative photo of a WT *D. sophia* and a T_1_ seedling with *DsPDS* edited. WT seedlings grew on 1/2 MS media, while mutant seedlings were screened from 1/2 MS media containing 50 mg/L of HygB. (**f**) The structure of *DsPDS* and the indel efficiency of *D. sophia* mutants analyzed by ICE. Coding sequences (CDS) of *DsPDS* are shown by blue boxes. The locations of sgRNA are illustrated by red triangles. (**g**) The editing profile of plant Sanger sequencing. The contributions show the inferred sequencing present in the edited population and their relative proportion. The cut site is represented by black dotted vertical line. The sgRNA target sequences are labeled by the black lines. The protospacer-adjacent motifs are labeled by red dotted horizontal lines.

In this study, the transformation in *D. sophia* was accomplished by dipping its buds for 45 s into *Agrobacterium* suspension, and the collected seeds were screened by selectable makers or molecular verification (Figure 1a and Figure S1). However, in planta transformation efficiency is influenced by several factors, such as the density of bacteria evaluated by optical density at 600 nm (OD_600_), application and concentration of acetosyringone (AS), concentration of surfactants, etc. [23]. To develop an effective and efficient floral dip transformation method for *D. sophia*, *A. tumefaciens* strain GV3101 containing pCAMBIA1301 was used to optimize the workflow with 18 treatments consisting of different variables (Figure 1b, Table S1). The result showed that a high *Agrobacterium* density (OD_600_ = 1.2) in the inoculation culture caused *D. sophia* flower wilting and death at any concentration of AS and Silwet L-77. It was consistent with the reports that high *Agrobacterium* density usually damaged the plant cells and resulted in lower cell recovery that ultimately reduced the stable transformation efficiency in *Pinus tabuliformis*, *Camellia sinensis*, spring bread wheat, etc. [23,24,25,26]. In addition, the transformation efficiency of OD_600_ = 0.6 was higher than that of OD_600_ = 0.3, illustrating that the optimal *Agrobacterium* density was OD_600_ of 0.6 (Figure 1b, Table S1).

Acetosyringone is usually employed to increase transformation efficiency by inducing *vir* genes expression to promote transfer and integration of *Agrobacterium* T-DNA into plant genomes [27,28,29]. Here, we evaluated the effect of AS at two concentrations (0 and 100 μM) on *D. sophia* transformation efficiency, showing that the inoculation solution without addition of AS resulted in a higher transformation rate than that of the culture with 100 μM AS (Figure 1b, Table S1). It was notable that with 100 μM AS application, the transformation efficiency dramatically reduced from 1.521% to 0.143% at the condition of OD_600_ = 0.6 and 0.03% Silwet L-77 (Figure 1b, Table S1). However, it was reported that in *Tamarix hispida*, 120 μM AS is the most suitable concentration for genetic transformation, as this treatment could significantly induce the expression of all *vir* genes in *A. tumefaciens* strain EHA105 with higher expression level except *virE2*, compared with other AS concentrations (0, 100, 150, 200 μM) [29]. It suggested that the optimal concentration of AS for *Agrobacterium*-mediated transformation could be affected by many factors, such as explant species and type, *Agrobacterium* strain. It may be worth confirming whether low concentration (<100 μM) of AS in *A. tumefaciens* GV3101 inoculation solution could improve *D. sophia* transformation efficiency in the future. The result in this study illustrated that no AS application in the *A. tumefaciens* inoculation solution may produce more transgenic seedlings.

Silwet L-77 is a low toxic surfactant that can reduce surface tension, which can improve the penetration of *Agrobacterium* to affect the transformation efficiency [15,30]. Many studies have found that appropriate dosage of Silwet L-77 can significantly increase the transformation rate in plant, such as *Linum usitatissimum*, *Cosmos sulphureus* Cav, Geng/*Japonica* and Xian/*Indica* rice [31,32,33]. To detect the effect of Silwet L-77 in *D. sophia* transformation, three different concentrations (0.03%, 0.05%, and 0.10% *v*/*v*) were assessed in this study. The result illustrated that the transformation efficiency (1.521%) with 0.03% Silwet L-77 was highest with OD_600_ = 0.6 and without AS (Figure 1b, Table S1). However, the efficiency dramatically reduced to 0.174% and 0.204% with the Silwet L-77concentration of 0.05% and 0.10%, separately (Figure 1b, Table S1). Thus, 0.03% Silwet L-77 was most effective in the floral dipping of *D. sophia*, and excessive Silwet L-77 could cause deleterious effects for *D. sophia* (Figure 1b, Table S1).

Although the floral dip method is a common technique of plant transformation, it has been mainly developed for some species belonging to the Brassicaceae family. Many factors more than those mentioned above have been suggested to impact the efficiency and application of the floral dip method in other plant species, such as physical barriers associated with flower morphology, necrotic reaction to the presence of *Agrobacterium* causing abortions in the flowers, low seed set, and big size of the plant and/or flower structures [34].

In summary, the floral dip method for *D. sophia* was most efficient when the flower buds were dipped into *Agrobacterium* suspension with OD_600_ = 0.6 and containing 0.03% Silwet L-77 for 45 s.

Various genetically encodable reporters have been widely used in transformation studies to monitor gene expression, protein localization, hormonal signaling, and other cellular activities [35,36,37]. In this study, Green fluorescent protein (GFP) and β-Glucuronidase (GUS) were utilized to confirm whether GFP and GUS could be acted as reporters in *D. sophia*. The HygB-resistant seedlings transformed with pCAMBIA1301 were verified by GUS staining assay. The result showed that strong GUS signals were observed in transgenic plants, using the WT as the control (Figure 1c). In addition, HygB-resistant seedlings infected with *Agrobacteria* carrying pCAMBIA1301-GFP were constructed to detect GFP in this study. The fluorescence microscope analysis demonstrated that transgenic seedlings showed strong green fluorescence, compared with WT (Figure 1d). In this study, it was validated that both GFP and GUS worked well in *D. sophia* and could be applied as reporters of *D. sophia* in the future studies.

CRISPR/Cas9 system-mediated gene editing is a powerful tool for gene function analysis and generation of elite germplasm. Additionally, phytoene desaturase (*PDS*) is involved in the carotenoid biosynthesis pathway, and the loss of function mutant of *PDS* genes shows obvious albino phenotype, prompting it to be widely used as an indicator for genome editing [38,39,40,41]. To detect whether the CRISPR/Cas9 system works well in *D. sophia*, the binary vector pHSE401-sgDsPDS carrying two sgRNAs (sg1 and sg2) targeting *DsPDS* gene was transformed into *D. sophia* by the floral dip method developed in this study. After antibiotic selection, a total of 36 HygB-resistant seedlings were obtained, and five of them were albino (Figure 1e).

Aiming to detect mutations generated by CRISPR in *D. sophia*, partial sequences of *DsPDS* gene, covering two sgRNA sites, were amplified from genomic DNA of albino seedlings. Sequencing results were further analyzed by ICE (ice.synthego.com (accessed on 7 September 2024)) to quantify the efficiency of CRISPR/Cas9 gene editing. Analysis of ICE showed that the editing efficiencies by CRISPR/Cas9 were 94–99% in both sgRNA1 and sgRNA2 of *DsPDS*, mainly contributed by the insertion event (Figure 1f and Figure S2). This insertion was mostly observed as a single-nucleotide insertion at each 3-bp upstream of the two PAMs in albino seedlings (T or A at sgRNA1 and T at sgRNA2), compared with WT (Figure 1g and Figure S2). For example, the mutant #4 showed 94 knockout score with indel of 94% in both sgRNA1 and sgRNA2 sites (Figure 1f,g). In sgRNA1 site, there were very clear deletions (18%) and insertions (76%) with a *p* value of <0.001 (Figure 1g). Whereas, only insertion (94%) was observed in sgRNA2 site with a *p* value of <0.001 (Figure 1g). In addition, substitutions were not detected in the genotyped plants, which might be caused by the low number of mutants used for sequencing. Finally, the targeted genome editing mediated by CRISPR/Cas9 system was successfully established in *D. sophia*.

## 3. Materials and Methods

### 3.1. Plant Materials and Growth Conditions

*D. sophia* used in this study was kept in our laboratory. The seeds were surface-sterilized with 70% ethanol for 30 s and 10% NaClO for 10 min, then rinsed three times in sterile water and dried on sterilized filter paper. The treated seeds were sown on half-strength Murashige and Skoog (1/2 MS) plates containing 0.8% agar and 2% sucrose and then kept in the artificial climate chamber (22 °C, under a 16 h day/8 h night photoperiod) for germinating. After one week, the young seedlings were transplanted to 15 cm diameter plastic pots with a 3:1 mixture of sterilized soil and vermiculite, at 28 °C, light/dark, 16/8 h with light intensity of 15,000 Lux in greenhouse conditions.

In about 30 days after sowing, *D. sophia* started to bolt and were ready for floral dipping (Figure 1a). The bolting plants carried plenty of young unopened flower buds and only a few open flowers. Notably, the buds for transformation should be at a relatively early stage of inflorescence development, when the oldest flower buds still have their petals mostly enclosed by sepals. These open flowers were excised before floral dipping. In this study, each individual plant of *D. sophia* represented a repeat in each treatment.

### 3.2. Vector Construction

A total of three vectors were used in this study. The T-DNA region of pCAMBIA1301 (Abcam, Cambridge, UK, Cat# ab275753) vector contains the Cauliflower Mosaic Virus 35S (CaMV 35S) promoter-driven *hygromycin phosphotransferase* II (*Hpt*II) gene, and the *GUS* gene as plant selection and reporter markers, respectively (Figure S3). In this vector, the *GUS* gene has a 5′ extension with a catalase intron to ensure expression in plants but not bacteria (Figure S3).

The pCAMBIA1301-GFP vector was constructed by replacing the GUS gene of pCAMBIA1301 with the GFP gene (Figure S3). In brief, the sequence of GFP was amplified with Nco-LP (5′-acgggggactcttgaccatggATGAGTAAAGGAGAAGAACTTTTCACTG-3′) Pml-RP (5′-gtcacctgtaattcacacgtgCTATTTGTATAGTTCATCCATGCCATG-3′) and inserted in the backbone of pCAMBIA1301 cut with *Nco*I and *Pml*I using a recombinant cloning kit (Vazyme, Wuhan, China, Cat#c-112).

The pHSE401 vector contains *gRNA*, *Cas9*, and *Hpt*II, a selectable marker [42]. *Cas9* is optimized by maize codon under the control of CaMV 35S promoter, and the *gRNA* is controlled by *Arabidopsis U6* promoter. The pHSE401-sgDsPDS plasmid was constructed based on pHSE401, which harbored two expression cassettes. The sgRNAs of *DsPDS* were designed using the online tool CRISPR-P (http://crispr.hzau.edu.cn/CRISPR2/ (accessed on 7 September 2024)) (listed in Figure 1f), and synthesized as oligos (Sangon, Shanghai, China), annealed, and cloned into pHSE401 vector.

### 3.3. A. tumefaciens-Mediated Delivery System of D. sophia

The GV3101 strain utilized in this study harbors the C58 chromosomal backbone containing rifampicin resistance and the Ti plasmid pMP90 (pTiC58DT-DNA) harboring the gentamicin resistance. The reporter and gene-editing vectors were transferred into *A. tumefaciens* strain GV3101 (pMP90) by the freeze/thaw method (keeping the mixture of vectors and *Agrobacterium* competent cells on ice for 30 min, liquid nitrogen for 5 min, 37 °C water bath for 5 min, back on ice for 5 min, separately). Colonies were selected with LB solid medium containing antibiotics (50 mg/L of rifampicin/Rif, 50 mg/L of kanamycin/Kan), and these positive colonies were dispersed into 50 mL LB liquid medium with 50 mg/L of Rif and 50 mg/L of Kan and cultured overnight (28 °C, 220 RPM). The overnight culture was pelleted by centrifuge and resuspended in 5% sucrose solution to a certain OD_600_ value. The transformation efficiency is influenced by several factors, such as the density of bacteria, concentration of AS and surfactants, etc. To establish the floral dip transformation method in *D. sophia*, we utilized three different OD_600_ of *Agrobacterium* culture, i.e., 0.3, 0.6, and 1.2. The suspension was added with varied concentrations of Silwet L-77 (0.03%, 0.05%, and 0.1% *v*/*v*) and AS (0 and 100 μM). A total of 18 treatments had been applied, and three plants were used in each treatment, each plant being a repeat. AS was added to *Agrobacterium* cell suspension 2 h before the floral dip, while the Silwet L-77 was added just prior to dipping. The *D. sophia* flower buds were dipped in *Agrobacterium* cell suspension for 45 s with a 50 mL falcon tube, and the dipped plants were wrapped with a plastic cover to maintain dark and high humidity for 12 h. Then, the treated plants were sent back to the normal growth conditions until the seeds were harvested. Collected seeds were surface-sterilized as mentioned above and germinated on 1/2 MS medium with 50 mg/L of HygB (Thermo-Fisher Scientific, Lenexa, USA, Cat#10687010) at 28 °C in the dark conditions for 5–7 days, to screen out the HygB-resistant seedlings. Then, these seedlings were transferred to wet soil and moved to the greenhouse at normal growth conditions for further verification.

### 3.4. GFP Detection and GUS Staining

The leaf and root of HygB-resistant seedlings transferred with pCAMBIA1301-GFP were applied to detect the fluorescence GFP gene, with WT as a control. The samples were visualized and imaged using an ultraviolet (UV) transilluminator (Olympus, Tokyo, Japan, BX53). HygB-resistant seedlings transferred with pCAMBIA1301 were stained according to the preparation protocol of GUS staining kit (Biosharp, Hefei, China, Cat#BL622A), with WT as a control. These samples were photographed under a microscope.

### 3.5. Evaluation of the Editing Efficiency in D. sophia

Genomic DNA was extracted from plant tissues with the CTAB method. To determine gene-editing type and efficiency, T_1_ transgenic plants transferred with pHSE401-sgDsPDS were analyzed by PCR amplification using the primers of PDS-F (5′-ATGGTTGTGTTTGGGAATG-3′) and PDS-R (5′-ATCATTGATCCCAAGTTCTC-3′), with WT as a control. The PCR products were separated by 1.0% agarose gel electrophoresis and sequenced (Sangon, Shanghai, China). The data obtained from Sanger sequencing were analyzed to determine the indel percentage and knockout score in genetically edited *D. sophia* through online software ICE (ice.synthego.com).

### 3.6. Statistical Analysis

Transformation efficiency = number of positive transgenic seedlings/total number of seeds × 100%. The data in Figure 1b are means ± standard deviations (SD), and the SDs were derived from three biological replicates.

## 4. Conclusions

In conclusion, a simple and efficient *Agrobacterium*-mediated genetic transformation system of medicinal and oilseed plant *D. sophia* was established via floral dip method. This method was accomplished by dipping the flower buds into *Agrobacterium* suspension with OD_600_ = 0.6 and containing 0.03% Silwet L-77 for 45 s. Moreover, the *DsPDS* gene was successfully edited by a CRISPR/Cas9 system that was developed in this study by *Agrobacterium*-mediated floral dip method. This genetic transformation and targeted editing system will be a valuable tool for routine investigation of gene function and further exploitation in *D. sophia*.

## Data Availability

The genomic sequence of the *DsPDS* genes have been deposited in the NCBI GenBank database with the accession number PP593058.

## Supplementary Materials

The following supporting information can be downloaded at: https://www.mdpi.com/article/10.3390/plants13202833/s1, Figure S1: Preliminary screening of T_1_ *D. sophia* plants on 1/2 MS media containing 50 mg/L HygB; Figure S2: Sequencing results of representative *DsPDS* knockout albino seedlings using CRISPR/Cas9 system, analyzed by ICE; Figure S3: The structure of the T-DNA region of pCAMBIA1301 and pCAMBIA1301-GFP. Table S1: Transformation efficiencies for *D. sophia* under different OD_600_ values of *Agrobacterium*, As concentrations and Silwet L-77 concentrations.
