# Supplementary material for: Establishment of a Genetic Transformation and Gene Editing Method by Floral Dipping in Descurainia sophia
Source: Plants (Basel). 2024 Oct 10;13(20):2833. doi: 10.3390/plants13202833 (PMC11510603; doi:10.3390/plants13202833)
Supplement: Supplementary file 1 [file plants-13-02833-s001.zip › plants-3223264-supplementary.pdf]

**Supplementary Materials:**

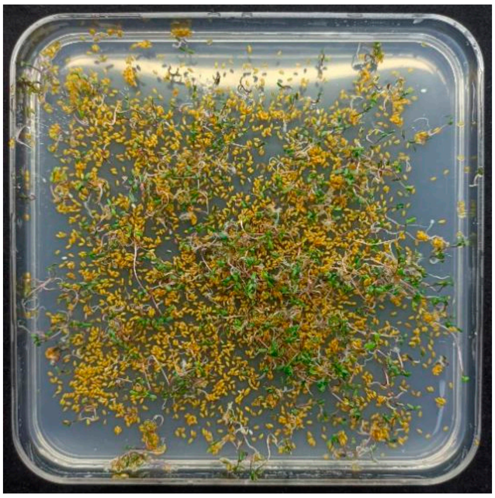

**Figure S1.** Preliminary screening of $T_1$ *D. sophia* plants on $^1/_2$ MS media containing 50 mg/L HygB.

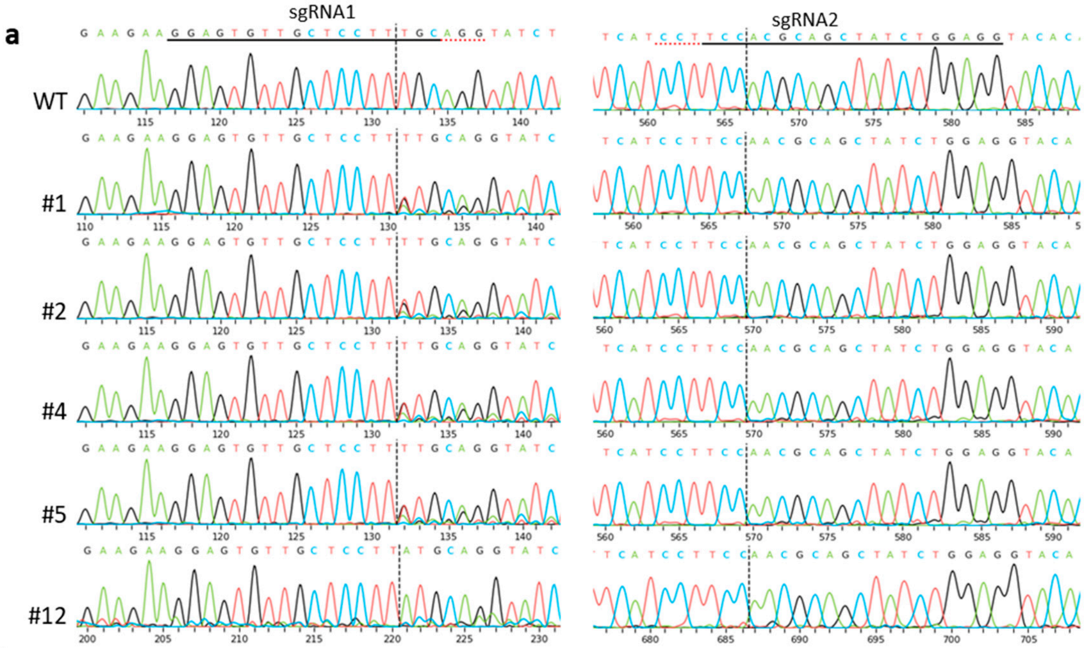


b

| Mutant | Indels | Indels |
|---|---|---|
| #1 | '1': 86.0%<br>'-1': 13.0% | '1': 99.0(%) |
| #2 | '1': 85.0%<br>'-1': 14.0% | '1': 99.0(%) |
| #4 | '0': 5.0%<br>'1': 76.0%<br>'-1': 18.0% | '0': 5.0(%)<br>'1': 94.0(%) |
| #5 | '0': 5.0%<br>'1': 76.0%<br>'-1': 18.0% | '0': 5.0(%)<br>'1': 94.0(%) |
| #12 | '1': 96.0(%) | '1': 98.0(%) |

'1': a single-nucleotide insertion; '-1': a single-nucleotide deletion; '0': off target editing

**Figure S2.** Sequencing results of representative *DsPDS* knockout albino seedlings

using CRISPR/Cas9 system, analyzed by ICE-SYNTHEGO. (a) Sequencing results of representative *DsPDS* knockout albino seedlings. The cut site is represented by black dotted vertical line. The sgRNA target sequences are labeled by the black lines. The protospacer-adjacent motifs are labeled by red dotted horizontal lines. (b) Efficiency of insertions and deletions in *DsPDS* knockout albino seedlings.

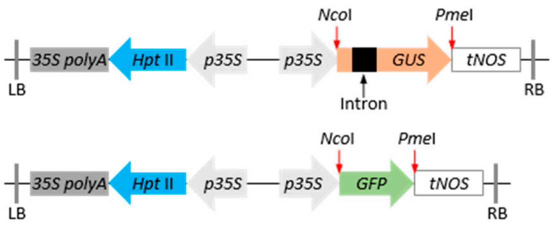


**Figure S3.** The structures of the T-DNA region of pCAMBIA1301 and pCAMBIA1301-GFP. They show the assembly of *Hpt* II gene expression cassette (*p35S*:*Hpt* II:*35S poly A*) and *GUS* gene expression cassette (*p35S*:*GUS*:*tNOS*). *Hpt* II, *hygromycin phosphotransferase* II gene; *GUS*, *β-glucuronidase* gene; GFP, *green fluorescent protein* gene; *p35S*, CaMV 35S promoter; *tNOS*, terminator of *nopaline synthase* (*NOS*) gene; *35S polyA*, CaMV poly A terminator; LB and RB: the left and right border of the T-DNA. Red arrows show the restriction sites utilized for pCAMBIA1301-GFP construction. Black box shows the intron in *GUS* gene.

**Table S1.** Transformation efficiencies for *D. sophia* under different $OD_{600}$ values of *Agrobacterium*, As concentrations and Silwet L-77 concentrations.

| $OD_{600}$ value of GV3101 | AS (μM) | Silwet L-77 (v/v)% | Number of Seeds | Number of Positive Transformed Seedlings | Transformation Efficiency (%) ± SD |
|---|---|---|---|---|---|
| 0.3 | 0 | 0.03 | 7400 | 5 | 0.070±0.018 |
| | | 0.05 | 9600 | 7 | 0.071±0.010 |
| | | 0.1 | 4520 | 0 | 0 |
| | 100 | 0.03 | 7350 | 0 | 0 |
| | | 0.05 | 4150 | 0 | 0 |
| | | 0.1 | 5700 | 5 | 0.091±0.003 |
| 0.6 | 0 | 0.03 | 10400 | 158 | 1.521±0.100 |
| | | 0.05 | 6900 | 12 | 0.174±0.012 |
| | | 0.1 | 4400 | 9 | 0.204±0.008 |
| | 100 | 0.03 | 6600 | 9 | 0.143±0.005 |
| | | 0.05 | 7900 | 6 | 0.081±0.001 |
| | | 0.1 | 8800 | 8 | 0.090±0.004 |
| 1.2 | 0 | 0.03 | 5000 | 0 | 0 |
| | | 0.05 | 5600 | 0 | 0 |
| | | 0.1 | 7200 | 0 | 0 |
| | 100 | 0.03 | 7600 | 0 | 0 |
| | | 0.05 | 8000 | 0 | 0 |
| | | 0.1 | 6900 | 0 | 0 |
